# APOE ε4-associated hippocampal atrophy trajectories across the Alzheimer's disease continuum: a systematic review, meta-analysis, and longitudinal validation

**DOI:** 10.3389/fnagi.2026.1847611

**Published:** 2026-07-01

**Authors:** Minnuo Cai, Hang Lei, Yuetong Zhang, Jiaxiang Zou, Wanjing Cao, Yiquan Wang, Kai Wei

**Affiliations:** 1 Xinjiang Key Laboratory of Biological Resources and Genetic Engineering, College of Life Science and Technology, Xinjiang University, Urumqi, Xinjiang, China; 2 Shenzhen X-Institute, Shenzhen, China; 3 College of Mathematics and System Science, Xinjiang University, Urumqi, Xinjiang, China

**Keywords:** AD spectrum, amyloid interaction, APOE-ε4, hippocampal volume, systematic review

## Abstract

**Background:**

Predicting patient-specific neurodegenerative trajectories is essential for targeted interventions in Alzheimer's disease. Although the APOE-ε4 allele is the predominant genetic risk factor for hippocampal atrophy, whether its structural impact reflects a static developmental phenotype or an accelerated neurodegenerative process conditional on amyloid pathology remains unresolved.

**Objectives:**

To quantify the effect of APOE-ε4 on hippocampal volume via cross-sectional meta-analysis, and to validate the gene-dose effect through independent longitudinal modeling in two cohorts.

**Eligibility criteria:**

Original observational neuroimaging studies reporting hippocampal volume stratified by APOE genotype in human participants across the Alzheimer's disease continuum.

**Information sources:**

PubMed, Embase, Web of Science, and Cochrane Library were searched from inception to August 2025.

**Risk of bias:**

Methodological quality was assessed using the Newcastle–Ottawa Scale (NOS) adapted for cross-sectional studies.

**Included studies and synthesis of results:**

The meta-analysis included 18 studies (*N* = 4, 311) and indicated reduced hippocampal volume in carriers (ICV-corrected stratum: SMD = −0.41, 95% CI [−0.67, −0.16], *p* = 0.004; *I*^2^ = 74.0%), with the effect absent in uncorrected analyses. Longitudinal gene-dose models, run independently in the post-QC NACC LMM sample (*N* = 3, 239; imaging extraction *N* = 3, 248) and ADNI (*N* = 1, 150) cohorts and combined via fixed-effect meta-analysis, demonstrated a dose-dependent acceleration of hippocampal atrophy: homozygotes exhibited a pooled additional volume loss of −58.25 mm^3^/year (95% CI [−89.26, −27.23], *p* = 2.33 × 10^−4^; *I*^2^ = 0%) and heterozygotes −34.32 mm^3^/year (95% CI [−52.31, −16.32], *p* = 1.85 × 10^−4^) relative to non-carriers.

**Limitations:**

Biomarker-stratified analyses used baseline-only CSF measurements subject to time-varying confounding and should be interpreted as exploratory. Moderate between-cohort heterogeneity was observed for the heterozygote effect (*I*^2^ = 63.0%).

**Conclusions and implications:**

These findings provide two-cohort evidence for a dose-dependent APOE-ε4 effect on hippocampal atrophy rates. Exploratory biomarker-stratified analyses in ADNI suggest this acceleration may be conditional on amyloid-β positivity, a hypothesis requiring validation with time-varying causal models.

**Systematic review registration:**

https://www.crd.york.ac.uk/PROSPERO/view/CRD420251243460, PROSPERO: CRD420251243460.

## 1 Introduction

Alzheimer's Disease (AD) is biologically defined by the accumulation of amyloid-β (Aβ) and tau proteins, and characterized by progressive hippocampal atrophy as a key marker of neurodegeneration (McKhann et al., 2011; Jack Jr et al., 2018; Braak and Braak, 1991). The ε4 allele of Apolipoprotein E (APOE-ε4) is the strongest genetic risk factor for late-onset AD (Corder et al., 1993; Farrer et al., 1997), yet its precise impact on hippocampal integrity remains unclear. In the current era of approved anti-amyloid monoclonal antibodies (e.g., lecanemab and donanemab), accurately predicting brain atrophy trajectories and stratifying patients for disease-modifying therapies has become increasingly important.

The nature of this genetic effect remains debated along several axes. Conflicting evidence exists regarding the timing at which APOE-ε4-associated hippocampal volume differences first emerge. The developmental hypothesis suggests a genetically determined structural difference present from early life (O'Dwyer et al., 2012; Knickmeyer et al., 2014), whereas the neurodegenerative hypothesis proposes accelerated decline in later life (Jack et al., 2010; Mormino et al., 2014; Raz et al., 2010). The allele dosage effect is similarly unresolved, as whether homozygous (ε4/ε4) carriers exhibit disproportionately severe atrophy compared to heterozygotes has not been fully characterized, leaving the linearity of genetic toxicity uncertain (Liu et al., 2013; Reiman et al., 2012). A further open question is whether APOE acts through direct structural toxicity (Montagne et al., 2020) or primarily as an upstream modulator dependent on amyloidosis (Mormino et al., 2014).

Evaluating the structural impact of APOE-ε4 is challenging because macroscopic volumetric assessments capture both genetically determined structural differences and the downstream effects of subclinical amyloid pathology, such as asymptomatic amyloidosis in clinically normal populations (Sperling et al., 2011; Vemuri et al., 2010). Methodological variations and demographic heterogeneity across studies further preclude a consensus effect size (Hibar et al., 2015).

Prior syntheses have addressed the association between APOE-ε4 and hippocampal volume but left critical questions unresolved. Liu et al. (2015) conducted a quantitative meta-analysis of 14 neuroimaging studies (*N* = 1,628) and reported a significant association between ε4 carrier status and reduced hippocampal volume; however, their analysis pooled studies irrespective of intracranial volume correction, potentially conflating genetic effects with uncontrolled head-size variation. Saeed et al. (2021) systematically reviewed 39 studies encompassing both cross-sectional and longitudinal designs and identified that longitudinal associations were more consistent than cross-sectional ones, yet the source of cross-sectional heterogeneity remained unidentified, motivating a call for well-powered, pathology-informed longitudinal investigations. More recently, Khan et al. (2026) demonstrated a dose-dependent interaction between APOE-ε4, baseline hippocampal volume, and the rate of cognitive decline in the ADNI cohort, suggesting that reduced hippocampal volume in carriers is associated with accelerated cognitive deterioration. However, their primary outcome was cognitive rather than structural, leaving unanswered the upstream question of whether and under what pathological conditions APOE-ε4 accelerates hippocampal atrophy itself. Longitudinal modeling of APOE-ε4-associated hippocampal atrophy using linear mixed-effects approaches has demonstrated gene-dose effects on atrophy rates (Hostage et al., 2013; Manning et al., 2014; Li et al., 2016); however, these analyses have uniformly relied on the ADNI cohort alone, limiting generalizability beyond a single research-enriched sample.

To address these questions, we adopted a progressive analytical strategy from cross-sectional meta-analysis to individual-level longitudinal validation. We first conducted a systematic meta-analysis stratified by intracranial volume correction status to establish a methodologically refined consensus effect size. We then analyzed two independent longitudinal cohorts differing in recruitment strategy, clinical composition, and imaging protocols. The NACC cohort, a large community-based clinical registry, provides the scale and heterogeneity necessary to characterize allele-dose effects on atrophy rates. The ADNI cohort, a highly characterized research cohort, enables both independent replication of the gene-dose effect and, through available CSF biomarker data, investigation of whether the structural impact of APOE-ε4 is conditional on the presence of amyloid pathology (Jack Jr et al., 2008; Beekly et al., 2007; Mormino et al., 2014). By running the same gene-dose model independently in both cohorts and combining results via fixed-effect meta-analysis, this design directly addresses the heterogeneity left unresolved by prior cross-sectional syntheses.

## 2 Methods

### 2.1 Meta-analysis strategy

**Figure 1:**
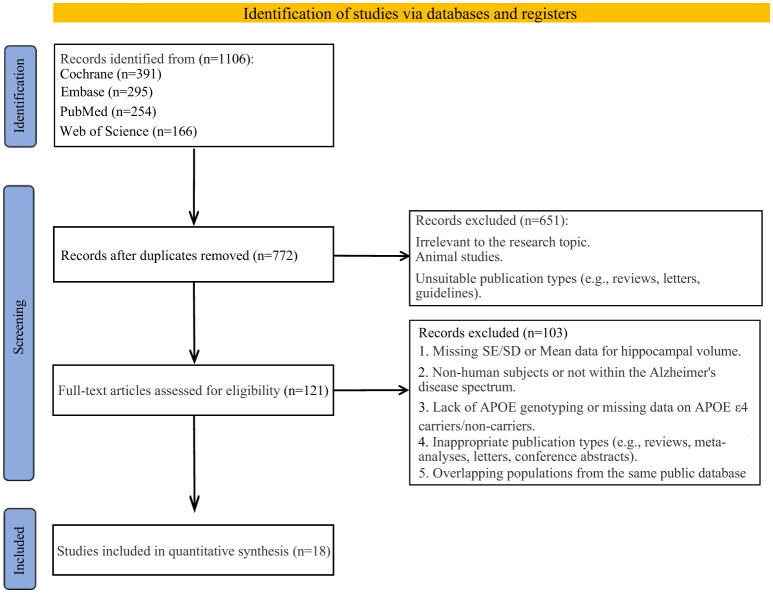
PRISMA flow diagram of the study selection process. The flowchart depicts the stepwise exclusion of records from identification to final inclusion. A total of 1,106 records were initially identified from four databases (Cochrane, Embase, PubMed, and Web of Science). Following duplicate removal, 772 records underwent title and abstract screening. Subsequently, 121 full-text articles were assessed for eligibility, with 97 excluded for not meeting the predefined inclusion criteria (see Supporting Information for details). Ultimately, 24 studies met all inclusion criteria. Of these, six were subsequently excluded during data preparation because five exhibited cohort overlap with other included publications and one had insufficient quantitative reporting, which yielded 18 independent studies for the quantitative meta-analysis (see Section 2.1 for details).

This systematic review and meta-analysis was conducted and reported in accordance with the PRISMA 2020 guidelines (Page et al., 2021) (Figure 1; the completed PRISMA 2020 checklist is provided in Table S1). We performed a systematic search of PubMed, Embase, the Web of Science Core Collection, and the Cochrane Library for original research published up to August 2025, following the Population, Intervention, Comparison, and Outcome (PICO) framework.

Data extraction was performed independently by two reviewers using a standardized form. Discrepancies were resolved through consensus discussion.

To prevent duplicate patient inclusion from shared databases and ensure cohort independence, we included only the most recent or largest publication for any given primary dataset. This step refined our initial selection from 24 eligible articles (*N* = 7, 326) to a final set of 18 unique studies (*N* = 4, 311). Four studies were excluded because their samples overlapped with the Alzheimer's Disease Neuroimaging Initiative (ADNI) cohort already represented by a larger retained publication (Khan et al., 2017; Hostage et al., 2013; Chiang et al., 2011; An et al., 2016); one was excluded for participant overlap with another study from the same clinical center (Pievani et al., 2011); and one was excluded because the original report provided only age ranges and non-significant trends without sufficient group-level statistics for effect-size calculation (Reiman et al., 1998) (Table 1). For the quantitative synthesis, we extracted sample sizes, means, and standard deviations of hippocampal volume for both APOE-ε4 carriers and non-carriers from these publications. We also recorded the hippocampal segmentation methodology employed by each study. Among the 18 included studies, eight used manual tracing protocols, six employed fully automated pipelines (e.g., FreeSurfer, SPM), and four used semi-automated approaches combining algorithmic segmentation with manual editing. To assess whether measurement methodology contributed to between-study heterogeneity, we performed a subgroup analysis stratified by segmentation method within the ICV-corrected stratum.

**Table 1:** Characteristics of the 18 studies included in the quantitative synthesis.

References	Sample size	Age (mean ±SD)	Gender (% male)	Included diagnosis
Koivumäki et al. (2024)	59	67.6 ± 4.7	36.7	CN
Dong et al. (2019)	117	58.1 ± 6.1	38.5	CN
Bussy et al. (2019)	162	38.8 ± 11.7	39.5	CN
Chang et al. (2019)	97	71.5 ± 7.8	54.6	AD
Wang et al. (2019)	1347	73.6 ± 7.1	55.8	AD, MCI, CN
O'Dwyer et al. (2012)	44	26.8 ± 4.6	59.1	CN
Alexopoulos et al. (2011)	33	24.5 ± 3.6	36.4	CN
Westlye et al. (2011)	95	63.8 ± 7.2	35.8	CN
Pievani et al. (2011)	28	71.5 ± 9.0	28.6	AD
Adamson et al. (2010)	50	61.0 ± 6.7	82	CN
Cherbuin et al. (2008)	331	62.6 ± 1.4	53.5	CN
Lind et al. (2006)	60	66.0 ± 8.1	38.3	CN
Lemaitre et al. (2005)	750	69.4 ± 2.9	42	CN
Den Heijer et al. (2002)	428	72.3 ± 7.0	47.8	CN
Geroldi et al. 1999)	58	70.7 ± 8.7	27.6	AD, CN
Plassman et al. 1997)	20	62.5 ± 7.8	30	CN
Lehtovirta et al. 1995)	42	69.4 ± 7.5	47.6	AD, CN
Soininen et al. 1995)	32	69 ± 6	31.3	CN

**Figure 2:**
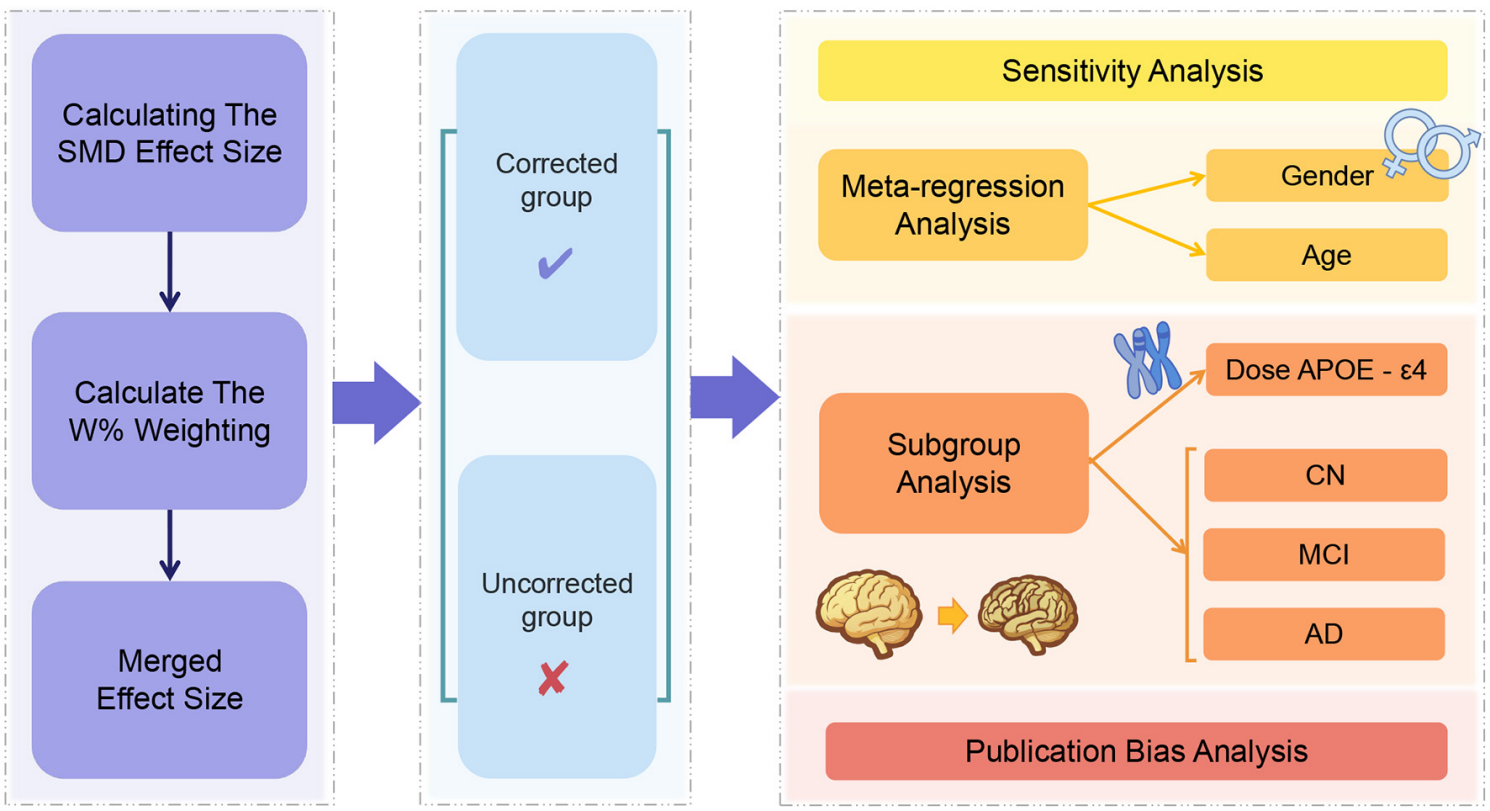
Flowchart of the meta-analysis procedure. Individual study SMDs were calculated and weighted (W%) to generate pooled effect sizes. Data were categorized into corrected and uncorrected groups. Analyses included sensitivity analysis, meta-regression (gender, age), subgroup analysis by APOE-ε4 dosage and diagnosis (CN, MCI, AD), and publication bias assessment.

The effect size was calculated as the Standardized Mean Difference (SMD) using Hedges' g. A random-effects model, with the between-study variance estimated using Restricted Maximum-Likelihood (REML), was employed to pool effect sizes (DerSimonian and Laird, 1986). The Hartung-Knapp adjustment was applied for more robust confidence intervals. Heterogeneity was quantified using the *I*^2^ statistic (Higgins and Thompson, 2002). To investigate sources of heterogeneity, we first performed subgroup analyses based on clinical diagnosis, APOE genotype dosage, and ICV correction methods (Higgins et al., 2003). Second, we conducted univariable random-effects meta-regressions to assess the potential moderating effects of mean participant age and sex distribution (Figure 2).

The methodological quality of each included study was assessed using the Newcastle–Ottawa Scale (NOS) adapted for cross-sectional studiesWells et al. (2000). Two reviewers independently scored each study across three domains (selection, comparability, and outcome); disagreements were resolved by consensus. Studies scoring ≥7 were considered high quality, 5–6 moderate quality, and ≤ 4 low quality. Individual study scores are reported in Table S3.

We performed a leave-one-out sensitivity analysis. Baujat plots were generated to visually identify studies contributing most to overall heterogeneity and the pooled effect. We implemented a multiverse analysis to test whether the main conclusion was robust to different analytical choices regarding outlier removal. Publication bias was assessed using Egger's regression test and the trim-and-fill method was used to estimate a bias-corrected effect size (Egger et al., 1997). Cross-sectional meta-analyses were performed in R (version 4.5.2) using the meta package (version 8.2.1) for effect-size pooling, heterogeneity quantification, and meta-regression (Balduzzi et al., 2019). Longitudinal linear mixed-effects models and two-cohort fixed-effect meta-analyses were implemented in Python (version 3.12.9) using the statsmodels package (version 0.14.6) (Seabold and Perktold, 2010).

Formal certainty-of-evidence assessment (GRADE) was not performed. The primary objective of the meta-analytic component was to quantify effect sizes and characterize sources of heterogeneity in the existing literature, thereby motivating the subsequent longitudinal validation rather than informing clinical guideline recommendations. The resolution of the identified heterogeneity was addressed directly through individual-level longitudinal modeling in two independent cohorts.

### 2.2 Longitudinal cohorts and participants

We used longitudinal data from two independent multicenter cohorts: the National Alzheimer's Coordinating Center (NACC) and the Alzheimer's Disease Neuroimaging Initiative (ADNI).

The NACC Uniform Data Set was used to assess the gene-dose effect in a large-scale clinical population (Beekly et al., 2007; Besser et al., 2018). Using the dataset from the September 2025 data freeze (covering visits between September 2005 and September 2025), we identified an imaging extraction of 3,248 participants with 4,007 observations, available pre-processed hippocampal volume measurements, confirmed APOE genotyping, and complete core covariates. After the residual-based cross-sectional QC step used for Model 1, 11 observations from 9 subjects were excluded, yielding 3,239 subjects and 3,996 observations for the final LMM sample.

The ADNI dataset served two complementary roles, namely independent replication of the gene-dose effect identified in NACC and biomarker-stratified analysis of the APOE-ε4–atrophy relationship (Mueller et al., 2005; Weiner et al., 2017). From the data downloaded in August 2025, we identified 1,150 participants who had longitudinal hippocampal volume measurements and confirmed APOE genotyping. For the gene-dose replication (Model 1), all 1,150 subjects were eligible regardless of CSF availability. For the biomarker-stratified analyses (Models 2 and 3), the subset with baseline Cerebrospinal Fluid (CSF) biomarker levels (Aβ42, p-Tau181, and t-Tau) was used, where baseline was defined as the earliest imaging visit of each participant. No raw MRI images were processed in this study; all volumetric data were obtained directly from the study repositories.

To ensure cohort independence, cross-verification of unique identifiers confirmed no overlapping subjects between the NACC and ADNI datasets.

Baseline demographic and clinical characteristics of both cohorts, stratified by APOE-ε4 dosage, are presented in Table 2. In both cohorts, homozygous carriers were enriched for Alzheimer's disease diagnosis relative to non-carriers (NACC *p* = 1.20 × 10^−26^; ADNI *p* = 1.57 × 10^−21^), supporting the need for baseline diagnostic adjustment in the longitudinal models.

**Table 2:** Baseline demographic and clinical characteristics of the longitudinal cohorts, stratified by APOE-ε4 dosage.

Variable	Non-carrier	Heterozygote	Homozygote	*p*-value
	(ε4 = 0)	(ε4 = 1)	(ε4 = 2)	
Panel A: NACC Cohort (*N* = 3,248; 4,007 observations)				
Age, years (mean ± SD)	68.3 ± 8.3	67.4 ± 8.2	65.5 ± 8.7	5.01 × 10^−6^
Female, *n* (%)	1258 (61.2%)	601 (59.4%)	100 (55.9%)	0.293
Diagnosis, *n* (%)				3.62 × 10^−26^
CN	1557 (75.7%)	650 (64.2%)	87 (48.6%)	
MCI	402 (19.5%)	255 (25.2%)	52 (29.1%)	
AD	98 (4.8%)	107 (10.6%)	40 (22.3%)	
Longitudinal visits (mean ± SD)	1.2 ± 0.5	1.2 ± 0.5	1.3 ± 0.6	0.020
Subjects with ≥2 visits, *n* (%)	395 (19.2%)	186 (18.4%)	48 (26.8%)	
Follow-up, years (mean ± SD)^†^	1.7 ± 0.6	1.8 ± 0.6	1.8 ± 0.5	0.303
Panel B: ADNI Cohort (*N* = 1,150; 1,948 observations)				
Age, years (mean ± SD)	74.0 ± 7.2	72.9 ± 7.2	70.2 ± 6.8	1.18 × 10^−6^
Female, *n* (%)	286 (45.7%)	198 (47.8%)	44 (40.0%)	0.338
Education, years (mean ± SD)	16.2 ± 2.7	15.9 ± 2.8	15.9 ± 2.7	0.232
Diagnosis, *n* (%)				3.34 × 10^−23^
CN	270 (43.1%)	93 (22.5%)	7 (6.4%)	
MCI	292 (46.6%)	225 (54.3%)	63 (57.3%)	
AD	64 (10.2%)	96 (23.2%)	40 (36.4%)	
CDR-SB (median [IQR])	0.5 [0.0, 1.5]	1.5 [0.5, 3.0]	2.0 [1.0, 3.9]	6.15 × 10^−24^
MMSE (median [IQR])	29.0 [27.0, 30.0]	28.0 [25.0, 29.0]	26.0 [24.0, 28.0]	1.19 × 10^−18^
Longitudinal visits (mean ± SD)	1.7 ± 0.9	1.7 ± 1.0	1.6 ± 0.8	0.466
Follow-up, years (mean ± SD)^†^	2.7 ± 1.5	2.5 ± 1.4	2.2 ± 1.2	0.025

Continuous variables are summarized as mean ± SD or median [IQR] as appropriate. *p*-values are from one-way ANOVA (normally distributed continuous variables), Kruskal–Wallis test (skewed continuous variables), or χ^2^ test (categorical variables). ^†^Follow-up duration computed among subjects with ≥2 visits only in the input datasets (NACC: *n* = 628; ADNI: *n* = 539). The NACC imaging extraction contains 3,248 subjects and 4,007 observations; the post-QC NACC LMM sample used in Model 1 contains 3,239 subjects and 3,996 observations. The NACC imaging analysis dataset was extracted from the MRI processing module and contains volumetric measurements with core demographic covariates (age, sex, clinical diagnosis). Education and CDR-SB are collected in the NACC Uniform Data Set (UDS) clinical module but were not linked to the imaging extraction used in this study. For the ADNI cohort, all variables were available from the integrated ADNIMERGE dataset. NACC is a clinical registry without a fixed follow-up schedule; accordingly, the proportion of subjects contributing ≥2 visits to the longitudinal models is reported in lieu of conventional dropout rates.

### 2.3 Data preprocessing and quality control

For the NACC dataset, a dedicated preprocessing and quality control (QC) pipeline was applied. To prevent endogeneity issues arising from disease progression itself, the clinical diagnosis of each subject was locked to the status recorded at the earliest visit. A two-stage QC was then performed. For cross-sectional QC, we fitted a linear model of hippocampal volume adjusted for age, sex, eTIV, and baseline diagnosis, and observations with standardized residuals exceeding a conservative threshold of ±4 SD were excluded. For longitudinal QC, we calculated the annualized rate of volume change and removed trajectories with biologically implausible changes, such as an annual volume increase greater than 20% or a loss exceeding 30%. The age covariate was centered and eTIV was scaled to optimize model performance and interpretability.

To construct a robust dataset for longitudinal analysis, a systematic preprocessing and quality control (QC) pipeline was applied to the ADNI data. To mitigate potential confounding from disease progression, clinical diagnosis was locked to the baseline status of each subject. The analysis was restricted to participants with at least two valid neuroimaging follow-up visits, ensuring the reliability of individual atrophy rate estimation. We then identified and removed potential outliers by calculating the standardized Z-score for hippocampal volume and excluding observations where the absolute Z-score exceeded 4, a step intended to reduce potential interference from factors such as image segmentation errors. Following this filtering process, hippocampal volumes were re-standardized based on the final analytical sample for use in subsequent statistical models. Given the relatively small longitudinal homozygote subsamples (NACC *n* = 48; ADNI *n* = 50), the longitudinal QC criteria were applied uniformly to guard against individual segmentation outliers or biologically implausible scans disproportionately influencing interaction slope estimates. Unlike the NACC clinical registry, which aggregates data from heterogeneous clinical sites with variable imaging protocols and scan intervals, ADNI employs standardized acquisition protocols with built-in quality assurance procedures. Because of this higher baseline data quality, the rate-based longitudinal QC applied to NACC (excluding annualized volume changes exceeding +20% or −30%) was not required for the ADNI sample; indeed, no additional observations were flagged by the cross-sectional Z-score filter.

### 2.4 Neuroimaging and biomarker definitions

This study used pre-computed hippocampal volume data provided by the NACC and ADNI databases. According to the protocols of these repositories, volumetric measures were derived from high-resolution T1-weighted MRI scans using automated segmentation pipelines (primarily FreeSurfer) (Fischl, 2012; Jack Jr et al., 2008; Fischl et al., 2002). In both cohorts, the analyzed variable represents total (bilateral) hippocampal volume.

For the ADNI longitudinal dataset, images were acquired using both 1.5T and 3T scanners and processed using FreeSurfer versions v4.4, v5.1, or v6.0 depending on the study phase. Rather than extracting variables from the raw MR image analysis tables, we used the pre-compiled ADNIMERGE dataset, in which the variable Hippocampus represents total hippocampal volume derived from the UCSF FreeSurfer cross-sectional pipeline (sum of left and right hemispheres). Hippocampal volumes were adjusted for intracranial volume (ICV; ADNIMERGE variable ICV) to correct for inter-individual head size differences.

The NACC dataset exclusively used scans acquired at 3T, processed with the more recent FreeSurfer v7.2. Total hippocampal volume (Hippocampus) and estimated total intracranial volume (eTIV) were extracted from the NACC MRI processing module. NACC volumes were adjusted using eTIV.

For the ADNI cohort, biomarker positivity was classified using the specific cut-off values provided in the dataset, where Aβ42 < 976.6 pg/mL defines Amyloid+, p-Tau181 > 21.8 pg/mL defines p-Tau+, and t-Tau > 245 pg/mL defines t-Tau+.

### 2.5 Statistical models

Longitudinal changes in hippocampal volume were analyzed using Linear Mixed-effects Models (LMMs) (Laird and Ware, 1982; Cnaan et al., 1997). To account for within-subject correlations and individual heterogeneity in baseline volume and atrophy rates, all models included random intercepts and random slopes for time at the subject level. Because the time coefficient and all Time × covariate interaction terms are estimated from within-subject change, subjects contributing only a single observation constrain the intercept but do not inform slope estimation; the effective longitudinal sample therefore comprises only subjects with ≥2 visits.

*Model 1: Gene-Dose Effect (NACC and ADNI)*. To quantify the dose-dependent acceleration of atrophy associated with APOE-ε4 and to explore potential modulation by age or sex, we modeled the hippocampal volume trajectories as


$$\begin{array}{l} Vol_{ij}=\beta_{0}+\beta_{1}\text{Tim}{\text{e}}_{ij}+\beta_{2}\text{APOE}4_{i}\\ \;+\beta_{3}\left(\text{Tim}{\text{e}}_{ij}\times \text{APOE}4_{i}\right)\\ \;+\beta_{4}\left(\text{Tim}{\text{e}}_{ij}\times \text{Ag}{\text{e}}_{i}\right)+\beta_{5}\left(\text{Tim}{\text{e}}_{ij}\times \text{Se}{\text{x}}_{i}\right)\\ \;+\beta_{6}\left(\text{Tim}{\text{e}}_{ij}\times \text{D}{\text{x}}_{i}\right)\\ \;+{\text{Z}}_{ij}\gamma +\left(u_{0i}+u_{1i}\text{Tim}{\text{e}}_{ij}+\varepsilon_{ij}\right) \end{array}$$ (1)

Here, Vol_*ij*_ represents the hippocampal volume for subject *i* at time *j*. The coefficient β_3_ captures the additional annual atrophy rate specific to ε4 carriers relative to non-carriers. The interaction terms β_4_ and β_5_ test whether atrophy rates vary with age or differ by sex, respectively. The term β_6_ allows atrophy rates to differ across baseline diagnostic groups (MCI and AD relative to CN), thereby controlling for the enrichment of progressive diagnoses among ε4 homozygotes. The term **Z**_*ij*_***γ*** denotes the main effects of covariates, including Age, Sex, estimated Total Intracranial Volume (eTIV), and Clinical Diagnosis.

The same model specification was applied to the ADNI cohort to provide an independent replication. The ADNI model substituted ICV (scaled by 1/1000, equivalent to the eTIV scaling in NACC) for eTIV and additionally included MRI field strength (1.5T vs. 3T) as a categorical covariate with both a main effect and a Time interaction, to account for systematic volumetric differences across scanner platforms present in the ADNI multi-site design. All other terms, including Time × APOE4 dosage, Time × Age (centered), Time × Sex, and Time × baseline diagnosis, were identical across cohorts.

To quantify the combined evidence across cohorts, the Time × APOE4 interaction coefficients from NACC and ADNI were pooled using inverse-variance weighted fixed-effect meta-analysis. Heterogeneity was assessed via Cochran's *Q* statistic and the *I*^2^ index. To control for diagnostic confounding by design, Model 1 was also re-estimated in the subset of cognitively normal (CN) participants from the NACC cohort. In a separate sensitivity analysis, we repeated the cross-sectional meta-analysis excluding the single ADNI-derived study (Wang et al., 2019) retained in our 18-study pool, to verify that the aggregate conclusion was independent of the cohort also used for longitudinal validation.

*Model 2: Biomarker-Stratified Analysis (ADNI, Individual Models)*. Because CSF biomarkers (Aβ42, p-Tau181, t-Tau) were measured at baseline and modeled as static covariates, these analyses are subject to time-varying confounding. Biomarker levels change over the follow-up period and are themselves influenced by both APOE genotype and neurodegeneration (Hernán et al., 2000). The interaction coefficients should therefore be interpreted as associations rather than causal effects. To separately test for a synergistic interaction between APOE-ε4 and each core pathology in the ADNI cohort, we constructed a series of hierarchical models. The general form for a given biomarker (Bio_*k*_) is


$$\begin{array}{l} Vol_{ij}=\beta_{0}+\beta_{1}\text{Tim}{\text{e}}_{ij}+\beta_{2}\text{APOE}4_{i}+\beta_{3}\text{Bi}{\text{o}}_{ki}\\ \;+\beta_{4}\left(\text{Tim}{\text{e}}_{ij}\times \text{APOE}4_{i}\right)\\ \;+\beta_{5}\left(\text{Tim}{\text{e}}_{ij}\times \text{Bi}{\text{o}}_{ki}\right)\\ \;+\beta_{6}\left(\text{APOE}4_{i}\times \text{Bi}{\text{o}}_{ki}\right)\\ \;+\beta_{7}\left(\text{Tim}{\text{e}}_{ij}\times \text{APOE}4_{i}\times \text{Bi}{\text{o}}_{ki}\right)\\ \;+{\text{Z}}_{i}\gamma +\left(u_{0i}+u_{1i}\text{Tim}{\text{e}}_{ij}+\varepsilon_{ij}\right) \end{array}$$ (2)

Here, APOE4_*i*_ is a binary indicator for carrier status, and the three-way interaction coefficient β_7_ is the primary term of interest. The covariate vector **Z** includes the main effects of baseline age, sex, intracranial volume (ICV), MRI field strength (1.5T vs. 3T), and baseline clinical diagnosis (CN/MCI/AD), as well as the Time × baseline diagnosis interaction and all constitutive lower-order terms of the three-way interaction. To account for systematic differences in volumetric measurements across scanner platforms, field strength was modeled as a categorical covariate with both a main effect on baseline volume and an interaction with time, thereby allowing atrophy rate estimates to be adjusted for potential scanner-related bias. The inclusion of the Time × diagnosis interaction ensures that the biomarker three-way interaction is not confounded by differential atrophy rates across diagnostic groups.

*Model 3: Biomarker-Stratified Analysis (ADNI, Joint Model)*. To assess the relative influence of Aβ and p-Tau pathologies, we constructed a joint model that simultaneously included all lower-order terms and the three-way interactions for both pathways:


$$\begin{array}{l} Vol_{ij}=\beta_{0}+\beta_{1}\text{Tim}{\text{e}}_{ij}+\beta_{2}\text{APOE}4_{i}\\ \;+\beta_{3}\text{A}{\text{β}}_{i}+\beta_{4}\text{pTa}{\text{u}}_{i}\\ \;+\beta_{5}\left(\text{Tim}{\text{e}}_{ij}\times \text{APOE}4_{i}\right)\\ \;+\beta_{6}\left(\text{Tim}{\text{e}}_{ij}\times \text{A}{\text{β}}_{i}\right)+\beta_{7}\left(\text{Tim}{\text{e}}_{ij}\times \text{pTa}{\text{u}}_{i}\right)\\ \;+\beta_{8}\left(\text{APOE}4_{i}\times \text{A}{\text{β}}_{i}\right)\\ \;+\beta_{9}\left(\text{APOE}4_{i}\times \text{pTa}{\text{u}}_{i}\right)\\ \;+\beta_{A}\left(\text{Tim}{\text{e}}_{ij}\times \text{APOE}4_{i}\times \text{A}{\text{β}}_{i}\right)\\ \;+\beta_{T}\left(\text{Tim}{\text{e}}_{ij}\times \text{APOE}4_{i}\times \text{pTa}{\text{u}}_{i}\right)\\ \;+{\text{Z}}_{i}\gamma +\left(u_{0i}+u_{1i}\text{Tim}{\text{e}}_{ij}+\varepsilon_{ij}\right) \end{array}$$ (3)

The covariate vector **Z** is identical to that in Model 2 (baseline age, sex, ICV, MRI field strength, and baseline diagnosis with Time interaction) and includes all constitutive lower-order terms. This model allows for a direct comparison of the interaction coefficients β_*A*_ and β_*T*_ after mutual adjustment.

## 3 Results

### 3.1 Establishing the baseline effect and sources of heterogeneity

**Figure 3:**
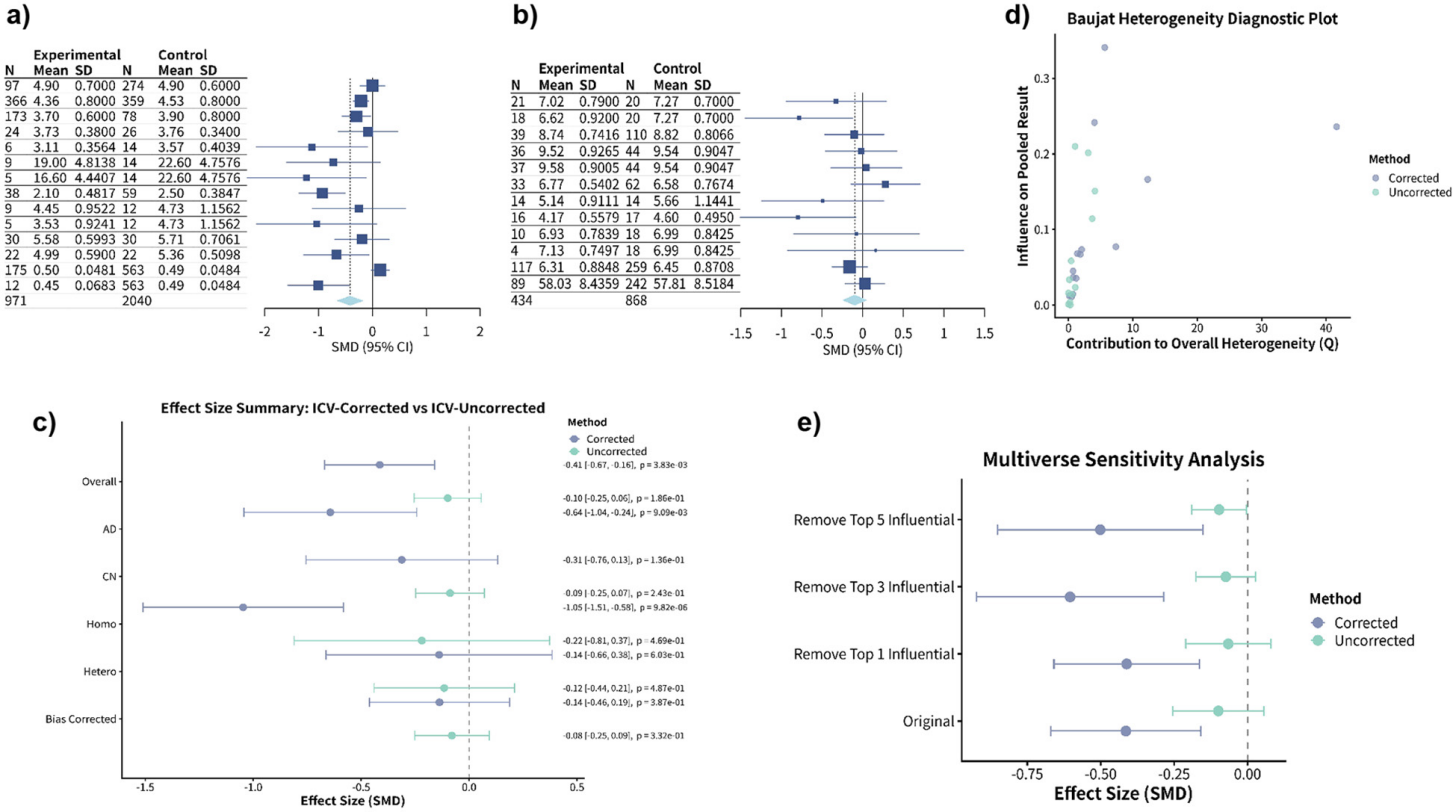
Meta-analytic results for APOE ε4 associated hippocampal volume reductions. **(a)** Forest plot of pooled SMD (Hedges' g) with 95% CIs for the ICV-corrected stratum (*k* = 14), stratified by clinical diagnosis. The overall SMD is −0.41 (95% CI[−0.67, −0.16], *p* = 0.004), the AD subgroup SMD is −0.64 (*p* = 0.009), and the homozygotes SMD is −1.05 (*p* = 9.82 × 10^−6^). **(b)** Forest plot for the uncorrected stratum (*k* = 12). The overall SMD is −0.10 (95% CI[−0.25, 0.06], *p* = 0.186). **(c)** Summary of subgroup and sensitivity effect sizes across corrected and uncorrected strata, including diagnosis-stratified, genotype-dosage, and bias-corrected estimates. **(d)** Baujat diagnostic plots identifying studies contributing disproportionately to overall heterogeneity and the pooled effect. In the corrected stratum, *I*^2^ = 74.0% and τ^2^ = 0.12. In the uncorrected stratum, *I*^2^ = 24.1% and τ^2^ = 0.002. **(e)** Multiverse sensitivity analysis after sequential removal of the top 1, 3, and 5 most influential studies. The corrected SMD ranges from −0.41 to −0.60, and the uncorrected SMD ranges from −0.07 to −0.10.

The quantitative synthesis included 18 eligible studies providing 26 distinct cohorts and a total of 4,311 participants. Risk of bias assessment using the Newcastle–Ottawa Scale indicated that all 18 included studies were of high methodological quality (NOS range: 7–9; median: 8; Table S3). The analyses were stratified based on whether the studies applied an intracranial volume (ICV) correction, a key methodological distinction. Among the 14 ICV-corrected cohorts, the random-effects meta-analysis revealed a reduction in hippocampal volume for APOE-ε4 carriers compared to non-carriers (Standardized Mean Difference [SMD] = -0.41, 95% CI [-0.67, -0.16], *p* = 0.004), accompanied by substantial heterogeneity (*I*^2^ = 74.0%, τ^2^ = 0.123). In contrast, among the 12 uncorrected cohorts, the effect was small and non-significant (SMD = -0.10, 95% CI [-0.25, 0.06], *p* = 0.186; *I*^2^ = 24.1%, τ^2^ = 0.002). This divergence indicates that ICV correction is a key methodological factor influencing the observed effect. Subsequent subgroup analyses are therefore based on the corrected stratum (Figure 3; Tables 1 and Table 3).

**Table 3:** Summary of main meta-analysis results.

Analysis	k	SMD	95% CI	*P*-value	*I* ^2^	τ^2^
Methodological stratification						
Corrected	14	−0.41	[−0.67, −0.16]	0.004	74.0%	0.123
Uncorrected	12	−0.10	[−0.25, 0.06]	0.186	24.1%	0.002
Clinical diagnosis (corrected only)						
AD	6	−0.64	[−1.04, −0.24]	0.009	45.3%	0.076
CN	7	−0.31	[−0.76, 0.13]	0.136	75.2%	0.154
Genotype dosage (corrected only)						
Homozygotes (ε4/ε4)	3	−1.05	[−1.51, −0.58]	9.82 × 10^−6^	0%	0
Heterozygotes (ε3/ε4)	3	−0.14	[−0.66, 0.38]	0.603	54.2%	0.122
Segmentation method (corrected only; *Q*_between_ = 0.58, *p* = 0.749)						
Manual tracing	5	−0.52	[−1.06, 0.02]	—	10.3%	0.038
Automated	6	−0.33	[−0.82, 0.15]	—	86.2%	0.171
Semi-automated	3	−0.52	[−1.75, 0.71]	—	48.0%	0.115

k, number of cohorts; SMD, Standardized Mean Difference (Hedges' *g*); 95% CI, 95% Confidence Interval; *I*^2^, percentage of variability due to heterogeneity; τ^2^, between-study variance. “Corrected” denotes studies adjusting for intracranial volume or total brain volume.

Stratification by clinical diagnosis within the corrected stratum indicated a gradient in the magnitude of APOE-ε4-associated atrophy. The association was most pronounced in patients with Alzheimer's Disease (*k* = 6), showing a volume reduction (SMD = -0.64, 95% CI [-1.04, -0.24], *p* = 0.009) with moderate heterogeneity (*I*^2^ = 45.3%). The effect in Cognitively Normal (CN) individuals (*k* = 7) was not statistically significant (SMD = -0.31, 95% CI [-0.76, 0.13], *p* = 0.136), yet it maintained considerable remaining heterogeneity (*I*^2^ = 75.2%). This high variability suggests the influence of unmeasured factors such as the varying prevalence of preclinical amyloid pathology across CN cohorts. A meta-regression within the CN subgroup yielded no evidence of an association between mean cohort age and effect size (*p* = 0.421). The inability of demographic proxies to explain this heterogeneity highlights a limitation of aggregate cross-sectional data and points to the necessity of individual-level biomarker profiling. Following the data processing protocol, only one cohort focused on Mild Cognitive Impairment (MCI) remained, which precluded a pooled subgroup estimate for this intermediate stage.

Further stratification within the corrected stratum identified a gene-dose effect. Homozygous (ε4/ε4) carriers exhibited a reduction in hippocampal volume (*k* = 3; SMD = -1.05, 95% CI [-1.51, -0.58], *p* = 9.82 × 10^−6^; *I*^2^ = 0%), whereas the effect in heterozygous (ε3/ε4) carriers was small and non-significant (*k* = 3; SMD = -0.14, 95% CI [-0.66, 0.38], *p* = 0.603; *I*^2^ = 54.2%).

A subgroup analysis stratified by hippocampal segmentation methodology within the corrected stratum indicated that measurement approach did not significantly moderate the pooled APOE-ε4 effect (*Q*_between_ = 0.58, *df* = 2, *p* = 0.749). The direction and magnitude of the association were consistent across manual tracing (*k* = 5; SMD = −0.52, 95% CI [−1.06, 0.02]; *I*^2^ = 10.3%), automated pipelines (*k* = 6; SMD = −0.33, 95% CI [−0.82, 0.15]; *I*^2^ = 86.2%), and semi-automated methods (*k* = 3; SMD = −0.52, 95% CI [−1.75, 0.71]; *I*^2^ = 48.0%). Although the overall genetic effect was robust across segmentation approaches, within-subgroup heterogeneity differed substantially. Studies employing manual tracing yielded highly consistent estimates (*I*^2^ = 10.3%), whereas those relying on fully automated pipelines exhibited considerable between-study variability (*I*^2^ = 86.2%), suggesting that algorithmic differences across software implementations contribute additional measurement variance beyond the biological signal.

Univariable meta-regressions within each stratum were performed to evaluate additional sources of heterogeneity. In the corrected stratum, neither the mean age of participants (*p* = 0.600) nor the proportion of female participants (*p* = 0.150) was a significant moderator. Similarly, in the uncorrected stratum, neither age (*p* = 0.622) nor sex (*p* = 0.895) was significant. Sensitivity analyses, including sequential removal of the most influential studies, showed that the overall conclusion remained stable. A trim-and-fill analysis of the corrected stratum suggested that smaller non-significant studies might be underrepresented. After imputing 6 studies, the bias-corrected effect size was attenuated (SMD = -0.14, 95% CI [-0.46, 0.19], *p* = 0.387). In the uncorrected stratum, only 1 study was imputed and the conclusion remained unchanged (SMD = -0.08, 95% CI [-0.25, 0.09], *p* = 0.332). The attenuation observed in the bias-corrected estimates and the reliance on cross-sectional data further emphasize the need for validation in longitudinal cohorts.

### 3.2 Longitudinal validation of the gene-dose effect

The meta-analysis indicated a potential non-linear pattern associated with gene dosage. Evaluating the NACC longitudinal cohort provided a means to test whether this cross-sectional observation reflects divergent individual atrophy trajectories over time. The NACC imaging extraction contained 4,007 observations from 3,248 subjects. After the residual-based cross-sectional QC step used for the LMM, Model 1 used 3,996 observations from 3,239 subjects, adjusted for baseline age, sex, eTIV, baseline clinical diagnosis, and their respective interactions with time (Table S4). Of these post-QC subjects, 628 (19.4%) contributed ≥2 longitudinal visits (mean 2.2 visits, range 2–5) and thus informed the slope estimates. The APOE-ε4 dosage distribution within this effective post-QC longitudinal subsample comprised non-carriers *n* = 395 (62.9%), heterozygotes *n* = 185 (29.5%), and homozygotes *n* = 48 (7.6%).

**Figure 4:**
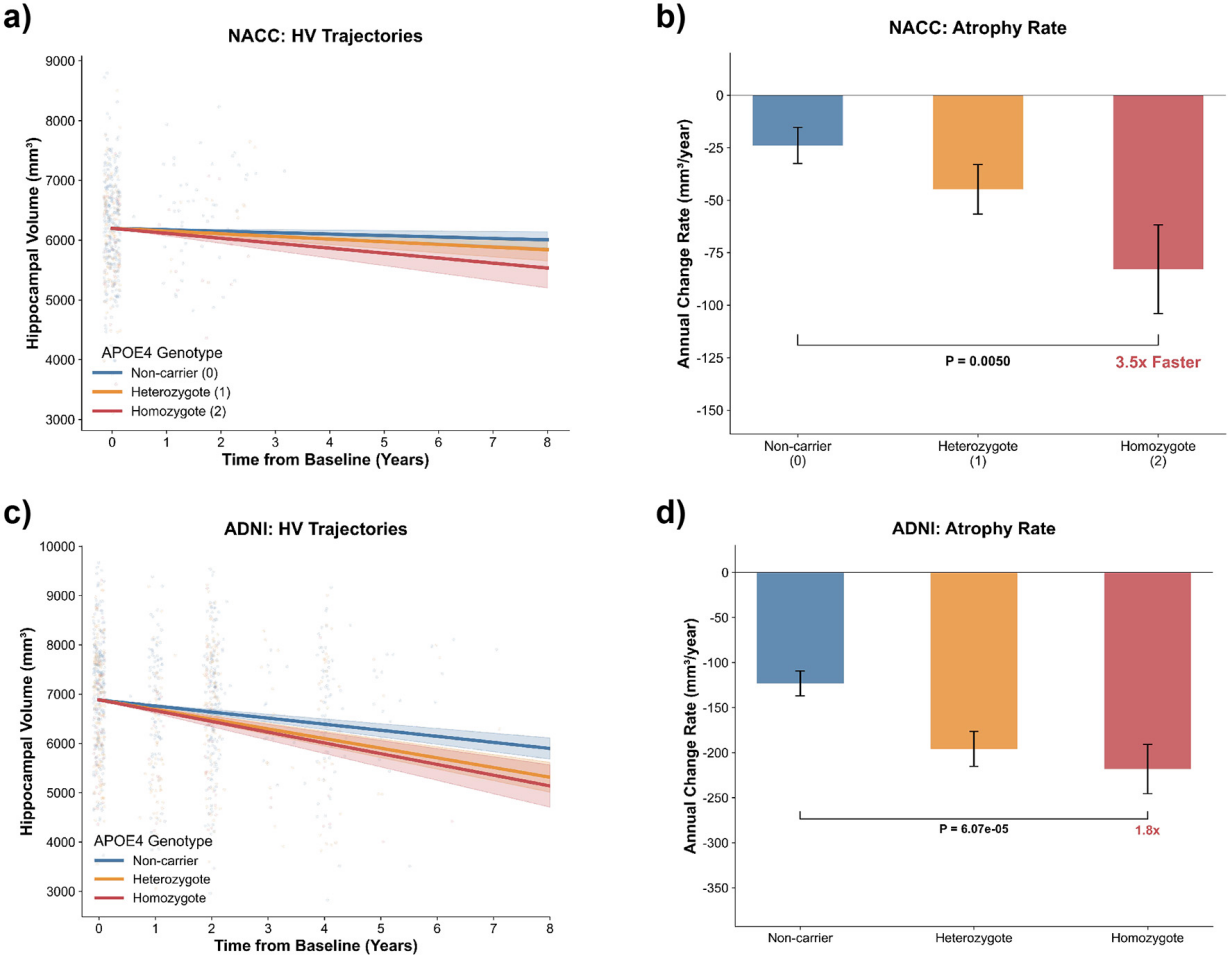
Longitudinal hippocampal atrophy trajectories and rates by APOE ε4 dosage. **(a)** Model-estimated longitudinal atrophy trajectories in the post-QC NACC LMM sample (*n* = 3, 239). Linear trend lines with 95% CIs are displayed for non-carriers, heterozygous, and homozygous carriers, adjusted for baseline diagnosis, age, sex, and eTIV. **(b)** Annual hippocampal atrophy rates by APOE ε4 dosage in the NACC cohort. Bars represent model-estimated annual volume change (mm^3^/year) with standard errors. **(c)** Model-estimated longitudinal atrophy trajectories in the ADNI cohort (*n* = 539). Linear trend lines with 95% CIs are displayed for non-carriers, heterozygous, and homozygous carriers, adjusted for baseline diagnosis, age, sex, ICV, and MRI field strength. **(d)** Annual hippocampal atrophy rates by APOE ε4 dosage in the ADNI cohort.

After accounting for differential atrophy rates across diagnostic groups (Time × AD: β = −95.35 mm^3^/year, *p* = 1.10 × 10^−4^; Time × MCI: β = −43.49, *p* = 0.001), non-carriers exhibited an estimated annual atrophy rate of −23.98 mm^3^/year (*p* = 0.005). The interaction between time and the homozygous genotype remained significant, corresponding to an additional volume loss of 58.96 mm^3^/year relative to non-carriers (Interaction β = −58.96, SE = 21.03, *p* = 0.005), indicating that the accelerated atrophy in homozygotes is not solely attributable to diagnostic composition (Figure 4).

The interaction term for heterozygous carriers indicated a non-significant additional decline of −20.79 mm^3^/year (*p* = 0.092). These longitudinal findings from an independent clinical cohort support the presence of a threshold-dependent gene-dose effect, where accelerated hippocampal decline is predominantly observed in the homozygous state.

To provide independent replication, the same gene-dose model was applied to the ADNI cohort. After quality control and restriction to subjects with ≥2 visits, the analytical sample comprised 539 subjects (1,337 observations; Non-carrier: *n* = 301; Heterozygote: *n* = 188; Homozygote: *n* = 50; mean follow-up: 2.59 years). Diagnostic groups exhibited significantly different atrophy rates (Time × AD: β = −132.62 mm^3^/year, *p* = 7.02 × 10^−6^; Time × MCI: β = −61.59, *p* = 4.35 × 10^−6^). After accounting for these differential rates, both APOE4 interaction terms remained significant: Time × Homozygote β = −57.32 mm^3^/year (SE = 24.04, *p* = 0.017) and Time × Heterozygote β = −51.14 mm^3^/year (SE = 13.75, *p* = 2.00 × 10^−4^). The non-carrier atrophy rate was −91.94 mm^3^/year (SE = 14.64, *p* = 3.38 × 10^−10^).

A fixed-effect meta-analysis pooling the Time × APOE4 interaction coefficients across both cohorts demonstrated a significant dose-dependent acceleration of hippocampal atrophy. For homozygotes, the pooled estimate was β = −58.25 mm^3^/year (95% CI [−89.26, −27.23], *p* = 2.33 × 10^−4^; Cochran's *Q* = 0.003, *I*^2^ = 0%). For heterozygotes, the pooled estimate was β = −34.32 mm^3^/year (95% CI [−52.31, −16.32], *p* = 1.85 × 10^−4^; *Q* = 2.70, *I*^2^ = 63.0%). The *I*^2^ = 0% for homozygotes indicates no evidence of between-cohort heterogeneity, though with only *k* = 2 studies the *Q* test has limited power and this should not be interpreted as proof of zero heterogeneity. The moderate heterogeneity observed for heterozygotes (*I*^2^ = 63.0%) reflects the divergence between NACC (non-significant) and ADNI (significant), a pattern discussed further below.

In a sensitivity analysis restricted to cognitively normal participants from the NACC cohort (*n* = 440 with ≥2 visits; Homozygote *n* = 20), the Time × Homozygote coefficient was directionally consistent (β = −54.44 mm^3^/year) though not statistically significant (*p* = 0.231), consistent with the limited statistical power of this subgroup.

As a sensitivity analysis, we repeated the cross-sectional meta-analysis after excluding the single ADNI-derived study (Wang et al., 2019) from the 14-study ICV-corrected pool. The corrected-stratum estimate remained significant (*k* = 11; SMD = −0.55, 95% CI [−0.88, −0.22], *p* = 0.004), indicating that the aggregate conclusion is independent of the cohort also used for longitudinal validation.

### 3.3 Exploratory biomarker-stratified analysis of the APOE-ε4 effect

**Figure 5:**
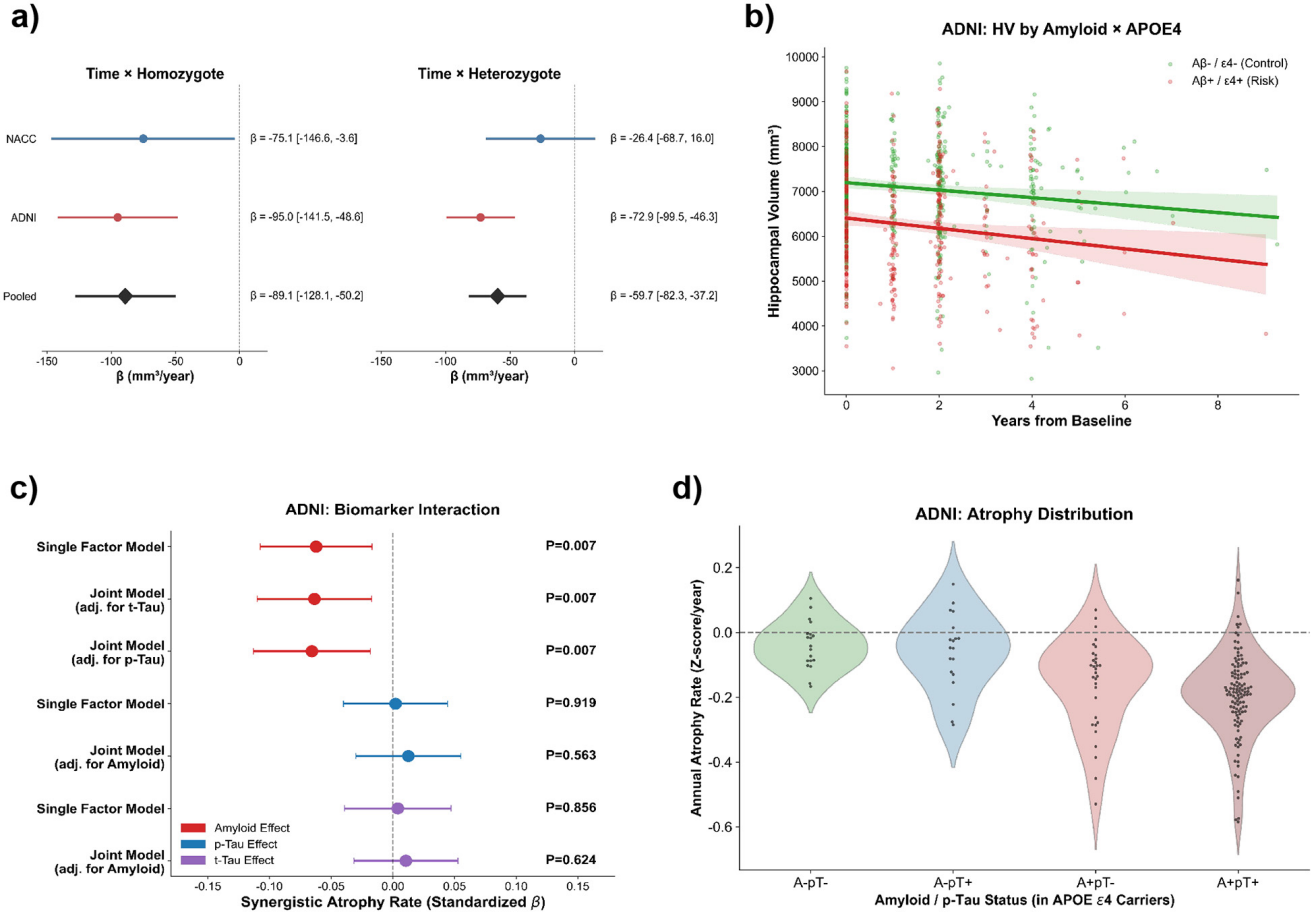
Cross-cohort synthesis and biomarker-stratified analysis of the APOE ε4 effect. **(a)** Fixed-effect meta-analysis forest plot pooling the Time × APOE4 interaction coefficients from the NACC and ADNI cohorts. The pooled homozygote estimate is β = −58.25 mm^3^/year (95% CI [−89.26, −27.23], *I*^2^ = 0%) and the pooled heterozygote estimate is β = −34.32 mm^3^/year (95% CI [−52.31, −16.32], *I*^2^ = 63.0%). **(b)** Longitudinal hippocampal atrophy trajectories stratified by amyloid and APOE ε4 status in the ADNI cohort, comparing the Aβ+/ε4+ group with the Aβ-/ε4- group with 95% confidence bands. **(c)** Forest plot of APOE ε4 × biomarker three-way interaction coefficients from linear mixed-effects models in the ADNI cohort (*n* = 539). The tested biomarkers include Aβ42, p-Tau181, and t-Tau, with the dashed line representing β = 0. **(d)** Distribution of individual-level annual atrophy rates in APOE ε4 carriers, stratified by amyloid and p-Tau status.

The unexplained variance in the cognitively normal meta-analysis subgroup and the non-linear effect observed in the NACC cohort suggest that the structural impact of APOE-ε4 may depend on specific pathological environments. The ADNI dataset enables evaluation of these biomarker interactions. Following quality control (initial sample 1,948 observations from 1,150 subjects; after excluding subjects with fewer than two visits 1,337 observations from 539 subjects across 1.5T [*n* = 557] and 3T [*n* = 780] scans), individual linear mixed-effects models tested the synergistic interactions between APOE-ε4 and core pathologies. The combination of APOE-ε4 and Aβ positivity was associated with a faster rate of hippocampal atrophy (standardized β = −0.06, *P* < 0.01). Synergistic interactions between the genotype and either p-Tau (*P* = 0.97) or t-Tau (*P*>0.99) were not statistically significant. A subsequent joint model including interaction terms for both Aβ and p-Tau confirmed that the Aβ interaction remained significant after adjusting for p-Tau (β = −0.07, *P* < 0.01), whereas the p-Tau interaction remained non-significant (*P*>0.5). The acceleration of neurodegeneration in APOE-ε4 carriers appears primarily associated with the amyloid pathway (Table 4, Figure 5).

**Table 4:** Biomarker-stratified interaction model results (ADNI).

Model	Marker	Coefficient	SE	*P*-value	*P*_*FDR*_
Single Factor Model	Aβ	-0.062	0.023	0.007	0.017
Single Factor Model	p-τ	0.002	0.022	0.919	0.919
Single Factor Model	t-τ	0.004	0.022	0.856	0.919
Joint Model (adj. for p-τ)	Aβ	-0.066	0.024	0.007	0.017
Joint Model (adj. for Amyloid)	p-τ	0.013	0.022	0.563	0.874
Joint Model (adj. for t-τ)	Aβ	-0.064	0.024	0.007	0.017
Joint Model (adj. for Amyloid)	t-τ	0.011	0.022	0.624	0.874

Coefficients represent the standardized three-way interaction term (Time × APOE4 × Biomarker). SE, standard error; *P*_FDR_, false discovery rate corrected *P*-value. “adj. for” indicates mutual adjustment in joint models.

This synergistic relationship between genotype and pathology corresponded with differences in clinical trajectories. Compared to Aβ-negative non-carriers (Aβ-/ε4-), the Aβ-positive APOE-ε4 carrier group (Aβ+/ε4+) exhibited a steeper decline in hippocampal volume over the longitudinal follow-up.

## 4 Discussion and conclusion

The structural impact of the APOE-ε4 allele on the hippocampus has long been debated, with conflicting evidence supporting either a lifelong developmental phenotype or an accelerated neurodegenerative process in later life. Our two-cohort longitudinal analysis provides evidence that APOE-ε4 homozygosity is associated with accelerated hippocampal atrophy, independently observed in both the NACC community-based registry and the ADNI research cohort. The consistency of the homozygote effect across cohorts differing in recruitment strategy, clinical composition, and imaging protocols (pooled β = −58.25 mm^3^/year, *I*^2^ = 0%) argues against single-cohort selection bias and supports a biological signal, extending prior single-cohort longitudinal demonstrations that were limited to the ADNI dataset (Hostage et al., 2013; Manning et al., 2014; Li et al., 2016). The gene-dose effect exhibits non-linearity, suggesting that homozygosity may represent a biological threshold rather than a simple additive risk (Castellano et al., 2011; Krasemann et al., 2017). Exploratory biomarker-stratified analyses suggest that this acceleration may be conditional on the presence of amyloid-β pathology, with carrier atrophy rates indistinguishable from non-carriers in the absence of amyloid. The between-cohort divergence in the heterozygote effect (*I*^2^ = 63.0%), which was significant in ADNI but not in NACC, is consistent with this amyloid-conditionality hypothesis. ADNI, enriched for MCI and AD participants, has a higher prevalence of amyloid positivity, potentially revealing a heterozygote effect that remains latent in the more clinically heterogeneous NACC sample.

This conditional relationship offers a potential explanation for the substantial heterogeneity observed in prior cross-sectional syntheses. Liu et al. (2015) reported high between-study heterogeneity (*I*^2^>70%) in their meta-analysis of APOE-ε4 and hippocampal volume without identifying its source. Our ICV-stratified analysis indicates that a substantial portion of this variability is attributable to inconsistent head-size correction across studies, as the genetic effect was significant only in ICV-corrected analyses. The conditional nature of the APOE-ε4 effect on atrophy also explains the pattern identified by Saeed et al. (2021), who noted that longitudinal associations were more consistent than cross-sectional ones. Cross-sectional samples inevitably combine amyloid-positive carriers experiencing accelerated atrophy with amyloid-negative carriers whose hippocampal trajectories are indistinguishable from non-carriers, thereby diluting the aggregate effect size. This interpretation reflects the aggregation bias inherent in evaluating genetic risk without individual-level biological staging. Our findings complement the recent work of Khan et al. (2026), who established that reduced hippocampal volume in APOE-ε4 carriers predicts faster cognitive decline within the ADNI cohort. By quantifying the upstream structural process, namely the amyloid-conditional acceleration of hippocampal atrophy, our two-cohort analysis identifies the mechanism that likely precedes the cognitive deterioration they observed, linking genotype to pathology to structure to clinical outcome.

Mechanistically, the biomarker-stratified analysis identified Aβ positivity, but not p-Tau or t-Tau, as the pathological context in which APOE-ε4 accelerates hippocampal atrophy. This specificity raises broader questions regarding the pathways mediating tissue loss. The non-significance of the p-Tau interaction may reflect the temporal decoupling between early fluid biomarker alterations and the accumulation of insoluble neurofibrillary tangles, which more directly correlate with regional atrophy (Barthélemy et al., 2020). Fluid biomarkers may therefore not fully capture the structural toxicity of aggregated tau, necessitating future validation with Tau-PET imaging (La Joie et al., 2020). APOE-ε4 may also exacerbate Aβ-associated structural toxicity through mechanisms outside the classical tau axis. Emerging evidence indicates that APOE-ε4 alters microglial lipid metabolism, shifting these cells toward a disease-associated microglia (DAM) phenotype (Koutsodendris et al., 2022). This innate immune response can promote neuroinflammation and subsequent neuronal damage, potentially operating in parallel with, or even preceding, extensive tau aggregation (Mormino et al., 2014; Jack Jr et al., 2013; Montagne et al., 2020). Consistent with this view, transcriptomic profiling in humanized APOE knock-in mice has shown that APOE-ε4 amplifies age-related brain expression changes beyond genotype-independent aging, implicating metabolic and synaptic pathways that may underlie the accelerated structural decline observed here (Labuza et al., 2025).

The pathology-dependent nature of APOE-ε4-associated atrophy has clinical implications, particularly in the context of emerging disease-modifying therapies. For Aβ-negative carriers, genetic risk should be decoupled from immediate neurodegenerative expectations, thereby refining prognostic counseling. Conversely, Aβ-positive homozygotes represent a population at elevated risk of structural decline. While these individuals exhibit a higher risk of amyloid-related imaging abnormalities (ARIA) during treatment with anti-amyloid antibodies (Van Dyck et al., 2023; Sims et al., 2023; Sperling et al., 2012), their accelerated natural disease course alters the risk-benefit calculus. Rather than routinely excluding homozygotes from therapeutic interventions, there is a rationale for investigating optimized dosing protocols to preserve structural integrity in this vulnerable subgroup.

Several methodological considerations warrant discussion. The included studies employed heterogeneous hippocampal segmentation approaches spanning manual tracing, semi-automated, and fully automated pipelines. Our subgroup analysis indicated that segmentation methodology did not moderate the pooled APOE-ε4 effect (*Q*_between_ = 0.58, *df* = 2, *p* = 0.749), suggesting that the overall conclusion is robust to measurement approach. However, within-subgroup heterogeneity differed substantially between methods, with minimal variance among manually traced studies (*I*^2^ = 10.3%) and considerable inconsistency among automated pipelines (*I*^2^ = 86.2%). This pattern suggests that cross-study variability in automated software implementations introduces methodological noise into aggregate estimates that is largely absent from standardized operator-guided protocols. This observation provides a complementary methodological rationale, alongside the biological rationale of unmeasured amyloid prevalence discussed above, for the longitudinal validation strategy adopted in this study. By analyzing cohorts processed through standardized, version-controlled FreeSurfer pipelines (NACC v7.2; ADNI v4.4–v6.0 within harmonized acquisition protocols), within-cohort measurement variance is minimized, enabling more precise isolation of the biological signal (Hedges et al., 2022). Furthermore, the hippocampus is not a molecularly homogeneous structure; recent single-population transcriptomic analyses have revealed distinct vulnerability signatures across CA1, CA3, and dentate gyrus neurons (Alldred et al., 2025), suggesting that subfield-level heterogeneity may contribute additional variance to aggregate volumetric measures.

The current analytical framework focuses on the primary Alzheimer's disease biomarkers and does not incorporate cerebrovascular contributions. Given that APOE-ε4 is independently associated with pericyte loss and blood-brain barrier dysfunction (Montagne et al., 2020), co-existing vascular pathologies likely provide synergistic contributions to tissue loss that remain unquantified here. While epidemiological evidence points to sex-based differences in APOE-ε4 risk (Farrer et al., 1997), aggregate-level meta-regressions lack the statistical power to reliably detect individual-level sex-by-genotype interactions.

The biomarker-stratified analyses (Models 2 and 3) used baseline CSF measurements modeled as static covariates. Because Aβ and tau are dynamic biomarkers that change over the follow-up period and are themselves influenced by both APOE genotype and neurodegeneration, this introduces time-varying confounding (Hernán et al., 2000). The interaction coefficients from these models should be interpreted as associations conditional on baseline biomarker status rather than causal effects. Formal causal inference regarding the mediating role of amyloid pathology would require marginal structural models or g-estimation applied to repeated biomarker measurements, which is beyond the scope of the present study.

The two-cohort meta-analysis revealed moderate heterogeneity in the heterozygote interaction (*I*^2^ = 63.0%), with the effect significant in ADNI but not in NACC. Several factors may contribute to this divergence. ADNI is enriched for MCI and AD participants relative to the community-based NACC registry, resulting in a higher prevalence of amyloid positivity potentially revealing a heterozygote effect otherwise diluted in clinically heterogeneous samples. Differences in mean follow-up duration, diagnostic ascertainment criteria, and imaging protocols between cohorts may also contribute. While this pattern is consistent with the amyloid-conditionality hypothesis, it cannot serve as causal evidence for it, and the limited number of cohorts (*k* = 2) precludes formal moderator analysis.

The two cohorts also exhibited substantially different non-carrier atrophy rates (NACC: −23.98 mm^3^/year; ADNI: −91.94 mm^3^/year). This approximately four-fold difference likely reflects the enrichment of ADNI for high-risk individuals who, even when cognitively normal at baseline, are more likely to harbor preclinical pathology, combined with differences in imaging acquisition (ADNI multi-site 1.5T/3T mixed protocols with FreeSurfer v4.4–v6.0 vs. NACC 3T-only with FreeSurfer v7.2) and clinical composition (ADNI: 43% CN, 47% MCI, 10% AD vs. NACC: 76% CN, 20% MCI, 5% AD). Despite these divergent baseline platforms, the APOE-ε4 homozygote additional atrophy rate was consistent across cohorts (−58.96 vs. −57.32 mm^3^/year; *I*^2^ = 0%), indicating that the genetic acceleration signal is robust to differences in absolute volumetric calibration.

In summary, this study provides two-cohort evidence for a dose-dependent APOE-ε4 effect on hippocampal atrophy rates, independently validated across community-based and research cohorts with distinct recruitment strategies and imaging protocols. Exploratory biomarker-stratified analyses suggest that this acceleration may be conditional on amyloid-β pathology, a hypothesis requiring formal validation with time-varying causal models. These findings suggest that precision prognostic models and future clinical trial designs may benefit from incorporating both allele dosage and amyloid staging to accurately predict individual structural trajectories across the Alzheimer's disease continuum.

## Data Availability

The code used for the meta-analysis and longitudinal modeling is publicly available in the GitHub repository: https://github.com/wyqmath/admeta.
