# Supplementary material for: APOE ε4-associated hippocampal atrophy trajectories across the Alzheimer's disease continuum: a systematic review, meta-analysis, and longitudinal validation
Source: Front Aging Neurosci. 2026 Jul 1;18:1847611. doi: 10.3389/fnagi.2026.1847611 (PMC13369312; doi:10.3389/fnagi.2026.1847611)
Supplement: Supplementary file 1 [file Data_Sheet_1.PDF]

Table S1: PRISMA 2020 Checklist.

#	Item	Reported on
**TITLE**		
1	Title	Title page
**ABSTRACT**		
2	Abstract	Abstract (structured per PRISMA 2020 for Abstracts checklist)
**INTRODUCTION**		
3	Rationale	Introduction, paragraphs 1–4
4	Objectives	Introduction, final paragraph
**METHODS**		
5	Eligibility criteria	Supporting Information §A.3
6	Information sources	Methods §2.1; Supporting Information §A.2
7	Search strategy	Table S2
8	Selection process	Supporting Information §A.2
9	Data collection process	Methods §2.1; Supporting Information §A.3
10	Data items	Supporting Information §A.3
11	Study risk of bias assessment	Methods §2.1; Table S3
12	Effect measures	Methods §2.1
13	Synthesis methods	Methods §2.1
14	Reporting bias assessment	Methods §2.1
15	Certainty assessment	Not applicable — justification in Methods §2.1
**RESULTS**		
16	Study selection	Results §3.1; Figure 1
17	Study characteristics	Results §3.1; Table 2
18	Risk of bias in studies	Results §3.1; Table S3
19	Results of individual studies	Results §3.1; Figure 3
20	Results of syntheses	Results §3.1; Table 3
21	Reporting biases	Results §3.1
22	Certainty of evidence	Not applicable
**DISCUSSION**		
23	Discussion	Discussion
**OTHER INFORMATION**		
24	Registration and protocol	Abstract (PROSPERO CRD420251243460)
25	Support	Declarations (Funding)
26	Competing interests	Declarations
27	Availability of data, code, and other materials	Declarations; GitHub repository

Table S2: Detailed Search Strategies for Electronic Databases (Search Date: August 2025)

Database	Search Strategy
**1. PubMed**	
#1	”Alzheimer Disease”[Mesh] OR ”Alzheimer*”[Title/Abstract] OR ”Alzheimer’s”[Title/Abstract] OR ”AD”[Title/Abstract] OR ”Senile Dementia”[Title/Abstract] OR ”Presenile Dementia”[Title/Abstract] OR ”Alzheimer Type Dementia”[Title/Abstract] OR ”FAD”[Title/Abstract] OR ”Familial Alzheimer Disease”[Title/Abstract] OR ”Early Onset Alzheimer*”[Title/Abstract] OR ”Late Onset Alzheimer*”[Title/Abstract]
#2	”Apolipoprotein E4”[Mesh] OR ”Apolipoprotein E”[Mesh] OR ”APOE”[Title/Abstract] OR ”APOE4”[Title/Abstract] OR ”ApoE-4”[Title/Abstract] OR ”epsilon 4”[Title/Abstract] OR ”e4 allele”[Title/Abstract] OR ”Apo E”[Title/Abstract]
#3	”Hippocampus”[Mesh] OR ”Hippocamp*”[Title/Abstract] OR ”Hippocampal Volume”[Title/Abstract] OR ”Hippocampal Atrophy”[Title/Abstract]
#4	”Magnetic Resonance Imaging”[Mesh] OR ”MRI”[Title/Abstract] OR ”Neuroimaging”[Title/Abstract] OR ”Voxel-based morphometry”[Title/Abstract] OR ”Volumetric”[Title/Abstract] OR ”Brain Volume”[Title/Abstract]
#5	#3 AND #4
#6	#1 AND #2 AND #5
**2. Embase**	
#1	’alzheimer disease’/exp OR ’alzheimer*’:ti,ab,kw OR ’senile dementia’:ti,ab,kw OR ’presenile dementia’:ti,ab,kw OR ’ad’:ti,ab,kw OR ’familial alzheimer disease’:ti,ab,kw
#2	’apolipoprotein E4’/exp OR ’apoe’:ti,ab,kw OR ’apoe4’:ti,ab,kw OR ’apolipoprotein e’:ti,ab,kw OR ’epsilon 4’:ti,ab,kw
#3	’hippocampus’/exp OR ’hippocamp*’:ti,ab,kw OR ’hippocampal atrophy’:ti,ab,kw OR ’hippocampal volume’:ti,ab,kw
#4	’nuclear magnetic resonance imaging’/exp OR ’mri’:ti,ab,kw OR ’magnetic resonance imaging’:ti,ab,kw
#5	#1 AND #2 AND #3 AND #4
**3. Web of Science**	
#1	TS=(”Alzheimer*” OR ”AD” OR ”Senile Dementia” OR ”Presenile Dementia” OR ”Alzheimer Type Dementia” OR ”FAD”)
#2	TS=(”Apolipoprotein E” OR ”APOE” OR ”APOE4” OR ”epsilon 4” OR ”Apo E4”)
#3	TS=(”Hippocampus” OR ”Hippocampal” OR ”Hippocampal Volume” OR ”Hippocampal Atrophy”)
#4	TS=(”MRI” OR ”Magnetic Resonance Imaging” OR ”Brain Volume”)
#5	#1 AND #2 AND #3 AND #4
**4. Cochrane Library**	
#1	MeSH descriptor: [Alzheimer Disease] explode all trees
#2	(Alzheimer* OR ”Senile Dementia” OR ”Presenile Dementia” OR ”Alzheimer Type Dementia” OR ”FAD” OR ”Familial Alzheimer Disease”):ti,ab,kw
#3	#1 OR #2
#4	MeSH descriptor: [Apolipoprotein E4] explode all trees
#5	MeSH descriptor: [Apolipoproteins E] explode all trees
#6	(APOE* OR ”Apolipoprotein E” OR ”Apolipoprotein E4” OR ”epsilon 4” OR ”e4 allele”):ti,ab,kw
#7	#4 OR #5 OR #6
#8	MeSH descriptor: [Hippocampus] explode all trees
#9	(Hippocamp* OR ”Hippocampal Volume” OR ”Hippocampal Atrophy”):ti,ab,kw
#10	#8 OR #9
#11	MeSH descriptor: [Magnetic Resonance Imaging] explode all trees
#12	(MRI OR ”Magnetic Resonance Imaging” OR ”Brain Volume” OR Volumetric OR ”Voxel-based morphometry”):ti,ab,kw
#13	#11 OR #12
#14	#3 AND #7 AND #10 AND #13

Table S3: Newcastle–Ottawa Scale (NOS) Quality Assessment of Included Studies (Adapted for Cross-Sectional Studies). Quality ratings: High ($\geq$7), Moderate (5–6), Low ($\leq$4). Selection domain assesses representativeness, sample size, ascertainment of exposure, and non-respondents. Comparability domain assesses control for confounders. Outcome domain assesses assessment method, statistical test, and data completeness.

Study	Selection (0–4)	Comparability (0–2)	Outcome (0–3)	Total (0–9)	Quality Rating
Adamson et al. (2010)	3	2	3	8	High
Alexopoulos et al. (2011)	3	1	3	7	High
Bussy et al. (2019)	4	2	3	9	High
Chang et al. (2019)	3	2	3	8	High
Cherbuin et al. (2008)	4	2	2	8	High
Den Heijer et al. (2002)	4	2	3	9	High
Dong et al. (2019)	4	2	3	9	High
Geroldi et al. (1999)	3	1	3	7	High
Koivumäki et al. (2024)	4	2	3	9	High
Lehtovirta et al. (1995)	3	1	3	7	High
Lemaître et al. (2005)	4	2	3	9	High
Lind et al. (2006)	4	2	3	9	High
O'Dwyer et al. (2012)	3	2	3	8	High
Pievani et al. (2011)	3	2	3	8	High
Plassman et al. (1997)	3	2	3	8	High
Soininen et al. (1995)	3	1	3	7	High
Wang et al. (2019)	4	2	3	9	High
Westlye et al. (2011)	3	2	3	8	High

Table S4: Full Results of the Linear Mixed-Effects Model on NACC Data after Residual-Based QC (3,239 Subjects; 3,996 Observations; with Time $\times$ Diagnosis Interaction)

**Predictor**	**Coefficient**	**Std. Error**	**z-value**	**P-value**	**95% Confidence Interval**
Fixed Effects					
Intercept	4199.09	100.89	41.62	$< 10^{-10}$	[4001.35, 4396.83]
APOE4 Dosage (Ref: Non-carrier)					
Heterozygote (1)	-99.62	26.79	-3.72	1.99×10^{-4}	[-152.12, -47.11]
Homozygote (2)	-336.14	54.94	-6.12	9.36×10^{-10}	[-443.83, -228.45]
Sex (Ref: Female) [Male]	306.01	29.43	10.40	2.48×10^{-25}	[248.33, 363.69]
Baseline Diagnosis (Ref: CN)					
AD	-706.55	47.48	-14.88	$< 10^{-10}$	[-799.62, -613.48]
MCI	-280.27	30.47	-9.20	3.58×10^{-20}	[-339.98, -220.55]
Time	-23.98	8.60	-2.79	0.005	[-40.84, -7.11]
Time $\times$ APOE4 Heterozygote	-20.79	12.33	-1.69	0.092	[-44.95, 3.38]
Time $\times$ APOE4 Homozygote	-58.96	21.03	-2.80	0.005	[-100.17, -17.75]
Time $\times$ Sex [Male]	-23.71	11.48	-2.07	0.039	[-46.20, -1.22]
Time $\times$ Baseline Diagnosis (Ref: CN)					
Time $\times$ AD	-95.35	24.66	-3.87	1.10×10^{-4}	[-143.67, -47.02]
Time $\times$ MCI	-43.49	13.67	-3.18	0.001	[-70.28, -16.69]
Age (Centered)	-24.38	1.48	-16.46	$< 10^{-10}$	[-27.28, -21.48]
Time $\times$ Age (Centered)	-2.98	0.71	-4.18	2.92×10^{-5}	[-4.38, -1.59]
eTIV (Scaled)	1.38	0.07	19.30	$< 10^{-10}$	[1.24, 1.52]
Random Effects Variance Components					
Group Variance	38.03				
Group $\times$ Time Covariance	0.03				
Time Variance	0.80				

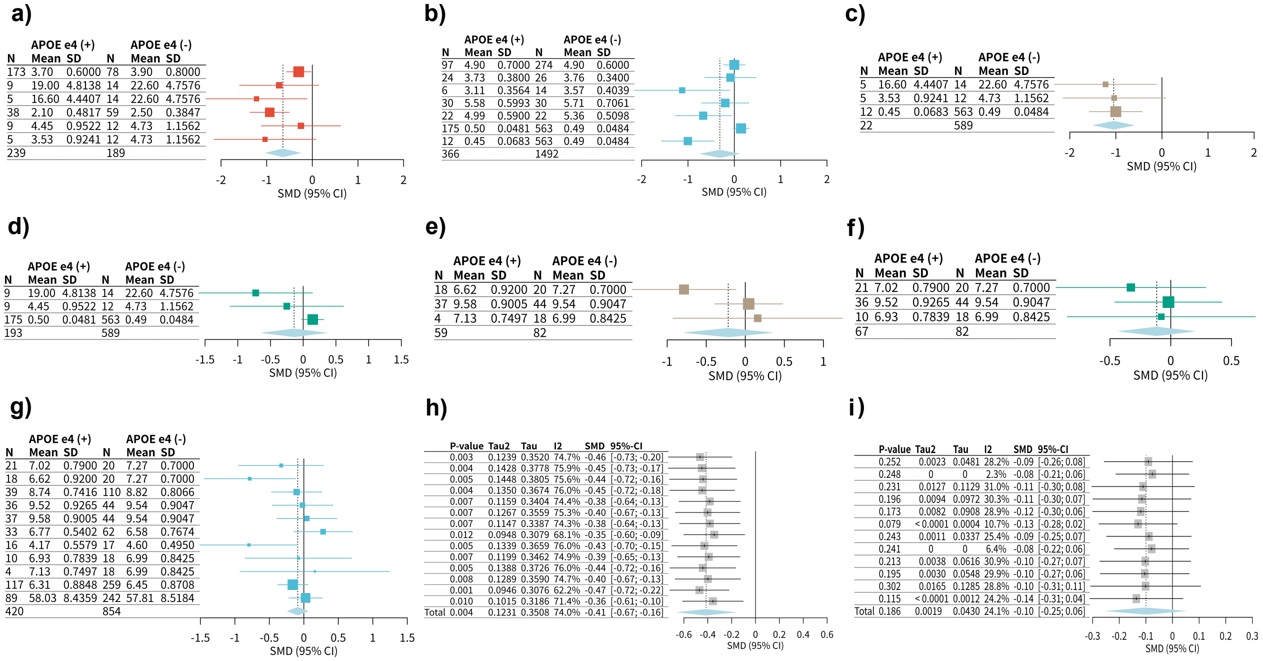

Figure S1: Supplementary forest plots and sensitivity analyses. (a) ICV-corrected forest plot, AD subgroup ($k = 6$; SMD $= -0.64$, 95% CI $[-1.04, -0.24]$, $p = 0.009$; $I^2 = 45.3\%$). (b) ICV-corrected forest plot, CN subgroup ($k = 7$; SMD $= -0.31$, 95% CI $[-0.76, 0.13]$, $p = 0.136$; $I^2 = 75.2\%$), indicating high residual heterogeneity not explained by demographic moderators. (c) ICV-corrected forest plot, ε4/ε4 homozygotes ($k = 3$; SMD $= -1.05$, 95% CI $[-1.51, -0.58]$, $p = 9.82 \times 10^{-6}$; $I^2 = 0\%$), demonstrating a large and consistent effect. (d) ICV-corrected forest plot, ε4 heterozygotes ($k = 3$; SMD $= -0.14$, 95% CI $[-0.66, 0.38]$, $p = 0.603$; $I^2 = 54.2\%$), indicating no significant cross-sectional effect for single-copy carriers. (e) Uncorrected forest plot, CN subgroup. (f) Uncorrected forest plot, ε4/ε4 homozygotes. (g) Uncorrected forest plot, ε4 heterozygotes. Panels (e)–(g) are consistent with the non-significant overall uncorrected stratum (SMD $= -0.10$, $p = 0.186$). (h) Leave-one-out sensitivity analysis, ICV-corrected method, confirming that no single study drives the pooled estimate. (i) Leave-one-out sensitivity analysis, uncorrected method, showing stable non-significance across iterations.

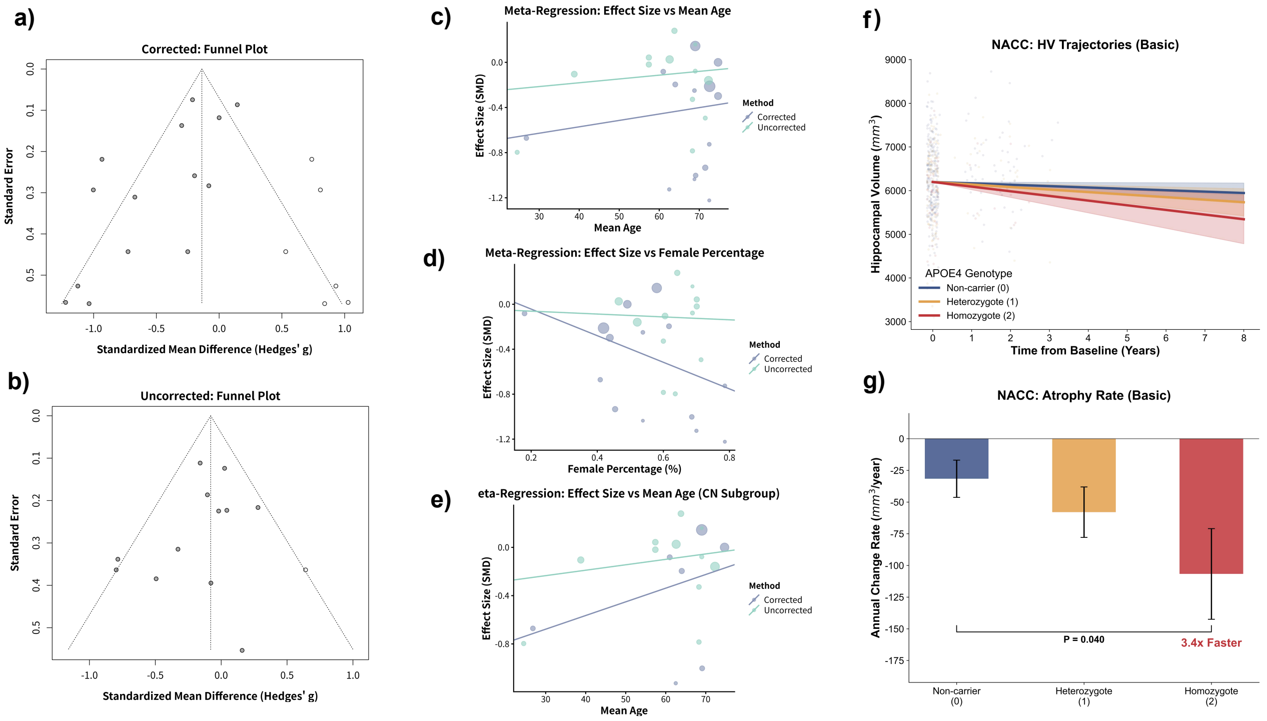

Figure S2: Supplementary meta-regression, funnel plots, and NACC longitudinal analyses. (a) Funnel plot with trim-and-fill, ICV-corrected stratum: 6 studies imputed, bias-corrected SMD $= -0.14$ (95% CI $[-0.46, 0.19]$, $p = 0.387$), suggesting potential publication bias attenuating the pooled estimate. (b) Funnel plot with trim-and-fill, uncorrected stratum: 1 study imputed, conclusion unchanged (SMD $= -0.08$, $p = 0.332$). (c) Meta-regression of effect size against mean age (corrected stratum $p = 0.600$; uncorrected $p = 0.622$), indicating age is not a significant moderator. (d) Meta-regression of effect size against female percentage (corrected $p = 0.150$; uncorrected $p = 0.895$), showing no significant sex-ratio moderation. (e) Meta-regression of effect size against mean age within the CN subgroup ($p = 0.421$), confirming that demographic proxies do not explain the residual heterogeneity. (f) NACC longitudinal atrophy trajectories from the basic model (without Time $\times$ Diagnosis interaction): non-carrier rate $= -31.61$ mm^3/year ($p = 0.031$), homozygote interaction $\beta = -75.11$ ($p = 0.040$), heterozygote $\beta = -26.37$ ($p = 0.223$). (g) NACC atrophy rates by APOE-ε4 dosage (basic model), illustrating a dose-dependent pattern that persists in the fully adjusted model (Table S4).
